# Cultural Identity and the Academic, Social, and Psychological Adjustment of Adolescents with Immigration Background

**DOI:** 10.1007/s10964-023-01853-z

**Published:** 2023-09-16

**Authors:** Jürgen Baumert, Michael Becker, Malte Jansen, Olaf Köller

**Affiliations:** 1 Max Planck Institute for Human Development, Berlin, Germany; 2 Technical University Dortmund, Dortmund, Germany; 3 Leibniz Institute for Research and Information in Education (DIPF), Frankfurt am Main/Berlin, Germany; 4 Institute for Educational Quality Improvement (IQB), Berlin, Germany; 5 Centre for International Student Assessment, Munich, Germany; 6 Leibniz Institute for Science and Mathematics Education (IPN), Kiel, Germany

**Keywords:** Cultural identity, Biculturalism, Immigrant youth, Developmental tasks, Adjustment

## Abstract

As Western societies become more ethnically and culturally diverse, understanding the acculturation of immigrant youth is essential for fostering social cohesion. How the cultural identity formation of ethnic minority adolescents relates to their academic, social, and psychological adjustment is an important and as yet unresolved research question. This study examined to what extent identifying with the heritage and/or host culture is an individual resource or risk factor for the adjustment of immigrant youth in Germany. A random sample of 15–17-year-olds (*N* = 1992; *M*_age_w1_ = 15.3 years, *SD* = 0.64; 44.5% girls; 44.7% students with immigrant background) was assessed twice: at the end of 9th and 10th grade. Academic performance and three dimensions of social/psychological adjustment (school attachment, self-esteem, and life satisfaction) were examined. Results showed that biculturalism was the modal identification pattern. Contrary to expectations, cultural identification did not differ systematically with perceived distance from the majority culture. Multivariate structural equation modeling revealed that both heritage and host identification can be developmental resources, but that their effects are dependent on the dimension of adjustment; biculturalism only proved to be a cumulative resource for school attachment. The domain specificity of the findings challenges the generalization claims of predominant acculturation theories.

## Introduction

Growing migration worldwide is one of the major challenges of the present time. Cultural diversity—once a feature of traditional immigration countries and former colonial powers—now characterizes almost all Western democratic states (OECD, 2022). It is only in recent decades that Germany has become a primary destination for global labor and refugee migration (McAuliffe & Triandafylidou, 2021). Today, it is a typical example of a host country in which increasing ethnic and cultural diversity at the individual level collides with institutional homogeneity and restrictive integration policies. How the conservative institutional context and the polarized debate on immigration affect the cultural identity formation and integration of immigrant youth is an important research question (De Coninck et al., 2021; Schwartz et al., 2018). This study examines patterns of cultural identification among adolescents with immigrant background in Germany, and tests whether identification differs based on region of origin and cultural distance from the majority society. It compares competing theories regarding the association between heritage/host identification and core dimensions of academic, social, and psychological adjustment. Finally, it tests whether the findings persist with control for a range of potential confounders at the individual and institutional level.

### Competing Theories of Cultural Identification and Adjustment

According to dual-identity theory, a bicultural orientation of immigrant youth (i.e., a strong identification with the culture of both the heritage and host societies) provides an optimal basis for successful development in adolescence and young adulthood, whereas marginalization (i.e., distance from both cultures) represents a developmental risk (Berry et al., 1989, 2006; Nguyen & Benet-Martínez, 2013; Meca et al., 2019, 2022; Schwartz & Unger, 2010). This theory is grounded in the assumption that identification with the heritage culture and identification with the host culture are both developmental resources that work in concert with additive effects—provided that the majority society condones dual self-categorization (Schwartz et al., 2018). Alternative models suggest that identification with a single culture might be sufficient for positive development. According to neo-assimilation theory, social and cultural assimilation is a long-term two-way process of weakening and shifting boundaries between minority groups and the majority society that nevertheless requires individual immigrants to adapt to core values, institutional arrangements, and cultural practices of the (changing) majority society if sociostructural integration and subjective well-being are to be achieved (Alba, 2008; Alba & Nee, 2003). In contrast, psychological critique of dual-identity theory and the associated Additive Resource Model suggests that secure identification with a single culture might be sufficient for sound personal identity development and psychological adjustment (Rudmin, 2009), and that identification with the heritage and host culture may be at least partially substitutable, as Ward and Kus (2012) have shown for agency-beliefs and Schotte et al. (2018) for self-esteem. These acculturation theories make relatively broad generalization claims that may not be justified if the adaptive functions of cultural orientations are in fact domain specific.

### Cultural Identification and the Societal and Institutional Diversity Climate

Young people growing up in immigrant families must learn to navigate at least two cultural contexts that are themselves subject to change. In elementary school, they become increasingly aware of cultural differences, and questions of group membership arise. During adolescence, they face the key developmental and acculturation task of exploring and defining their cultural and ethnic identity. Succeeding in this task is considered to be an important resource for accomplishing the generic developmental tasks faced by all young people (Meca, Allison, et al., 2023; Motti-Stefanidi et al., 2012; Motti-Stefanidi & Masten, 2020). According to acculturation research, whereas positive attitudes to immigration facilitate all types of identification (see, e.g., Schachner et al., 2018), negative attitudes—whether to immigration in general or to particular immigrant groups—can make it harder for young people from ethnic minorities to identify with the host society, instead promoting identification with the heritage culture (Rumbaut, 2008; Schwartz et al., 2018). Negative attitudes to immigration are apparent at the societal level in the form of xenophobia, restrictive immigration and integration policies, and negative stereotyping and rejection of immigrant groups. The MIPEX Index (https://www.mipex.eu/) and the European Social Survey (ESS; Heath & Richards, 2020) seek to capture such processes in international comparison. According to the ESS, acceptance of immigrant groups decreases with the perceived cultural distance from the host country. In Germany, it is highest for immigrants of the same ethnic background, decreases gradually across other European and non-European countries, and is lowest for Islamic countries. At the institutional level, negative attitudes are reflected in access to, participation in, and acceptance within societal institutions; at the behavioral level, they emerge as exclusion, discrimination, and microaggression. This study focuses on acceptance within educational institutions—in particular, on the diversity climate of schools.

Studies on the relationship between societal and institutional diversity climate and cultural orientations are scarce, and results are mixed. In a cross-country comparison, Yağmur and van de Vijver (2012) found that members of the Turkish minority identified more strongly with the host culture in immigration-friendly countries, but that their identification with the culture of origin did not differ notably across countries. Schachner et al. (2017) compared six countries with differing integration policies, expecting to find that both heritage and host identification facilitate integration in schools in immigration-friendly countries but that only host identification has a positive effect in hostile countries. In fact, the results tended to support the Additive Resource Model of biculturalism, with both identifications serving as a resource for integration irrespective of the societal context. Likewise, in comparisons across immigrant groups, both cultural identifications varied independently of the distance from the majority society (Jugert et al., 2020; Schachner et al., 2014).

At the institutional level, empirical findings on the relationship between the diversity culture of schools, cultural identity, and adjustment of immigrant students are likewise mixed. In a multilevel analysis, Schachner et al. (2016) found that host identification was higher and heritage identification was lower in schools with a diversity climate valuing equality and inclusion (egalitarianism); however, no associations emerged between cultural identification and multiculturalism. In a second study, Schachner et al. (2018) found that host identification was much higher in schools with a climate valuing both multiculturalism and egalitarianism, whereas heritage identification was not dependent on school climate. Overall, however, there is limited empirical support for the theoretically plausible assumption that the societal and institutional diversity culture of the host country directly shapes the cultural identity development of ethnic minority adolescents.

### Cultural Identification and Academic Adjustment

Most previous studies on the relationship between cultural identification and sociocultural adaptation in adolescence have focused on academic performance, usually measured in terms of school grades. Strong identification with the host culture tends to go hand in hand with greater familiarity with the country’s education system and greater willingness to invest in education (Alba, 2008). Host identification is therefore seen as an adaptive resource that helps students to succeed at school. The empirical findings are largely consistent across geographical areas and minority groups (Edele et al., 2013; Hannover et al., 2013; Kiang et al., 2013; Schachner et al., 2014; Schotte et al., 2018). The role of identification with the heritage culture seems more ambivalent: It can buffer the negative effects of discrimination (Eccles et al., 2006), but also amplify stereotype threat effects (Armenta, 2010). Whether it has direct effects on academic adjustment remains unclear. The findings reviewed by Rivas-Drake et al. (2014) and Makarova and Birman (2015) are inconsistent, but the authors highlight that positive effects of heritage identification may be mediated by enhanced self-esteem. A number of more recent studies have reported null findings or negative effects (Armenta, 2010; Edele et al., 2013; Hannover et al., 2013; Schachner et al., 2014; Schotte et al., 2018).

Following Berry et al. (1989), a separate group of studies has taken a typological approach to the relationship between bicultural orientation and adjustment. The meta-analysis by Nguyen and Benet-Martínez (2013) concluded that biculturalism is likely to be a general resource for academic and psychosocial adjustment, whereas distance from both cultures seems to pose a general risk. However, the results vary depending on how the typology was established (Arends‐Tóth & van de Vijver, 2007). Studies that follow Berry’s classification approach by dichotomizing and recombining the two identity scales have been criticized for assuming four classes a priori without being able to confirm the model fit; for failing to account for adolescents who are undecided and still exploring their social identity; and for setting cut-points arbitrarily (Rudmin, 2003, 2009; Schwartz et al., 2010; Schwartz & Zamboanga, 2008). More data-driven explorative studies using latent profile analyses tend to focus on dimensions of psychological adjustment. Only Wantchekon and Umaña-Taylor (2021) have addressed academic adjustment, reporting better grades for Black and Latinx students with secure ethnic identification than for those with diffuse ethnic orientation.

### Cultural Identification and School Attachment

School is one of the main socialization agencies in adolescence. To thrive, students need to both engage with curricular and extracurricular activities and enter into social relationships, thus developing motivational, social, and emotional attachment to school. School attachment, along with social networks, is seen as an important indicator of adolescents’ social integration in the proximal environment (Salmela-Aro & Upadaya, 2012). For ethnic minority students, developing school attachment means navigating multiple cultures. Biculturalism is likely to be a cumulative resource here. The few studies on these relationships have found that both heritage and host identification seem to support attachment to secondary school. Abu-Rayya and Sam (2017) reported positive correlations between biculturalism and school attachment for all but one of the 13 countries participating in the International Comparative Study of Ethnocultural Youth (ICSEY; Berry et al., 2006). Schachner et al. (2017) found similar results across six countries. Further support for this pattern of findings has come from Birman et al. (2010) for Russian immigrants to the United States, Kiang et al. (2013) for Asian Americans, and Horenczyk (2010) for Ethiopian and Russian immigrants to Israel.

### Cultural Identification and Psychological Adjustment

At its core, psychological adjustment means developing a sense of social and personal identity that affords self-acceptance, personal well-being, and life satisfaction. According to self-categorization theory, which focuses on the self and social group memberships (Turner & Reynolds, 2011), heritage identification and host identification should both enhance psychological adjustment, as the individual compares the respective reference group to the outgroup on dimensions that favor the reference group. Heritage identification can be expected to stabilize the self-esteem and life satisfaction of ethnic minority adolescents, especially if experiences of discrimination and negative stereotyping make it difficult for them to identify with the majority culture (Schmitt et al., 2014). The reviews by Rivas-Drake et al. (2014) and Makarova and Birman (2015) and the meta-analysis by Smith and Silva (2011) confirm that a strong heritage identity is indeed a resource for psychological adjustment. Recent studies have also shown that a secure heritage identity was able to buffer the negative psychosocial impact of racial discrimination against Asian immigrants in the United States during the COVID-19 pandemic (Litam & Oh, 2022; Oh et al., 2023). However, findings on the role of host identification are mixed. Dimitrova et al. (2015) reported null findings for the relationship between host identification and psychological adjustment in Turkish immigrants to Bulgaria and Germany; Kiang et al. (2013) had null findings in a sample of Asian Americans. In contrast, and in line with the Additive Resource Model of biculturalism, Birman et al. (2010) and Schachner et al. (2014, 2016) observed positive relationships with psychological adjustment for both orientations. Schotte et al. (2018) found two additive main effects for life satisfaction, but a small negative interaction for self-esteem, indicating that strong host identification may compensate for low heritage identification. Ward and Kus (2012) reported similar findings for agency beliefs. Shamloo et al. (2023) found that a bicultural identity can support subjective well-being and coping with stress even in extreme situations, such as the COVID-19 pandemic.

Most studies taking a typological approach by dichotomizing and recombining the two cultural orientation scales have reported positive effects of dual identification and supported, at least to some extent, the Additive Resource Model (see the meta-analysis by Nguyen & Benet-Martínez, 2013, and the review by Schwartz et al., 2018). However, studies with a person-centered approach using cluster or latent profile analyses have arrived at very different classifications (Jugert et al., 2020; Schwartz & Zamboanga, 2008; Spiegler et al., 2019; Zhang et al., 2018), especially when cultural identity was conceptualized multidimensionally or additional variables such as cultural practices or acculturation stress were used in the classification (Meca et al., 2015, Meca, Cruz, Lucero, et al., 2023; Meca, Cruz, Veniegas, et al., 2023). A recent study from Italy with sample of early adolescents (Karataş et al., 2023) is the only one to have reproduced Berry’s typology. However, most of these typological studies identified one or more classes of doubly identified individuals, with membership of this group being generally positively associated with dimensions of psychological adjustment. A shared weakness of these studies is that they explore identity configurations without being able to analyze the mechanisms of their interplay.

### Summary and Research Gaps

There is only limited empirical support for the idea that context factors contribute directly to individual identity development. Findings converge in showing that host identification is important for academic and social adjustment and that heritage identification is related to psychological adjustment, but whether and to what extent heritage identification also facilitates academic adjustment remains disputed. It also remains an open question whether the two identifications have cumulative effects on psychological adjustment, as interactions are generally not tested. For social adjustment, in contrast, the few findings on school attachment clearly confirm the Additive Resource Model of biculturalism. In sum, there is a need for research that investigates several adjustment dimensions simultaneously, examines the domain-specific additive and interaction effects of cultural identification, controls for immigrant generation, immigrant group membership, and their potential moderator function, and takes into account the cultural diversity climate of the school attended.

## Current Study

**Fig. 1:**
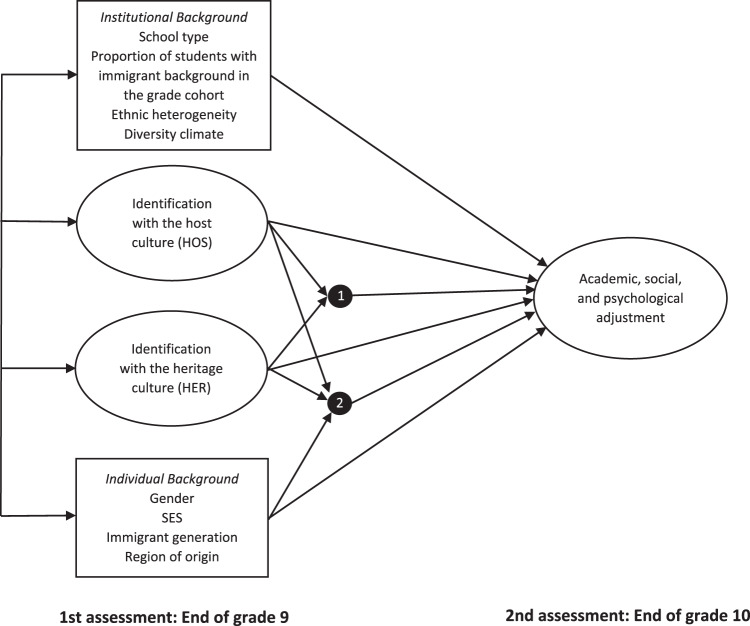
Theoretical model of the present investigation. Note. 1 = Interaction between HOS and HER. 2 = Interaction between HOS/HER and individual background

Understanding the acculturation of immigrant youth is essential for fostering social cohesion. Yet surprisingly little is known about the extent to which the social and institutional diversity climate of a host country and the perceived cultural distance between groups shape the cultural identity formation of ethnic minority adolescents. This study attempts to fill the knowledge gaps identified above in three steps. First, it examines patterns of cultural identification among ethnic minority adolescents in Germany and tests to what extent heritage and host identification and the probability of bicultural identification depend on the acceptance of the respective immigrant group in the host society. Second, it systematically investigates how the two cultural orientations are related to adolescents’ academic, social, and psychological adjustment. Third, it examines whether the findings persist with control for (a) immigrant generation, region of origin, and sociodemographic characteristics and (b) the institutional context of school, and particularly the normative diversity beliefs endorsed by majority group students. Figure 1 presents the theoretical model, which draws on the integrative risk and resilience model proposed by Suárez-Orozco et al. (2018). Based on previous findings, the following hypotheses were formulated: Systematic differences were expected in immigrant groups’ cultural identification at the societal level, with heritage identification increasing, host identification decreasing—and, accordingly, the probability of bicultural identification decreasing—as cultural distance between heritage and host society grows (Hypothesis 1a). At the institutional level of schools, a diversity climate in which majority group peers endorse multiculturalism was expected to facilitate both heritage and host identification (Hypothesis 1b). Host identification was expected to serve as a resource for academic performance at school, while heritage identification was expected to constitute a risk factor (Hypothesis 2a). Both orientations were expected to facilitate school attachment, providing further evidence for the Additive Resource Model of biculturalism (Hypothesis 2b). Likewise, both identifications were expected to be a resource for self-esteem and life satisfaction (Hypothesis 2c); no directed hypothesis about a possible interaction of the two orientations was formulated.

## Methods

### Database

This study draws on data collected as part of the BERLIN study on educational decisions and pathways in the 2013/2014 and 2014/2015 school years (Neumann et al., 2017). In the city state of Berlin, students are assigned to one of two secondary school tracks after 6 years of elementary education based on their performance and parental preferences. In 2014, 57% of the age cohort attended an Integrated Secondary School (ISS) and 43% an academic-track *Gymnasium* (GY) school. A two-stage disproportionate random sample was drawn for the BERLIN study. In the first step, *N* = 70 ISS and *N* = 29 GY were drawn; the probability of drawing a school was proportional to its size. All sampled schools participated in the survey; the school-level coverage rate was thus 100%. In the second step, a sample of *n* = 25 15-year-olds and *n* = 10 non-15-year-old 9th graders was randomly selected within each school, covering students both with and without an immigrant background. The baseline sample for the study was a stratified random sample of 2,109 grade 9 students. The sample was weighted on the base of registry data to compensate for disproportionate sampling. There were two waves of assessment: Wave 1 at the end of grade 9 in 2014 and Wave 2 at the end of grade 10 in 2015. Following the sampling procedures of the PISA study (OECD, 2019), all students who had attended a German school for less than one year or who were unable to understand the oral and written instructions and questionnaires presented in German were excluded from the study. The achieved sample—i.e., the students who participated in at least one wave—was *N* = 1992, amounting to a response rate of 94.5%. Panel mortality between Waves 1 and 2 was 14%, primarily due to class retention in ISS (see Table 1). The present study focuses on the *N* = 842 students identified as having an immigrant background.

**Table 1:** Sample characteristics by study wave

Characteristics	Participation in longitudinal study				
	Waves 1 and 2	Wave 1 only	Wave 2 only	Total	*F* test
*N* (%)^a^	1622 (81.4)	278 (14.0)	92 (4.6)	1992 (100)	–
Age (mean/*SD*)	15.4/0.64	15.7/0.82	15.5/0.71	15.4/0.67	**
Gender (% female)	48.3	48.2	43.0	48.0	ns
Socioeconomic status (HISEI, mean/*SD*)	57.6/19.8	54.7/18.1	–	57.4/19.7	*
Immigration background (%)^b^	43.6	32.4	–	42.5	*
Proficiency in German (mean/*SD*)^c^	0.08/0.95	−0.29/1.17	–	0.00/1.00	**
Cognitive abilities (mean/*SD*)^c^	0.07/0.98	−0.27/1.10	–	0.03/1.00	**

^a^Achieved sample

^b^At least one parent not born in Germany; reported in student or parent questionnaire

^c^WLE estimator, *z* standardized

**p ≤ 0.01, *p ≤ 0.05

### Measures

In Wave 1 at the end of grade 9, students were administered a reading comprehension test assessing their command of German and a questionnaire assessing family background variables and other individual characteristics (e.g., acculturation beliefs, command of the heritage language, heritage identification, host identification); parents completed a questionnaire on the family background and social situation. In Wave 2 at the end of grade 10, students completed a questionnaire tapping the four adjustment dimensions.

#### Cultural Identification

Identification with the heritage culture and identification with the host culture were assessed using an instrument from the German National Educational Panel Study (NEPS; https://www.neps-data.de). Each cultural identification was assessed by four items, based on the Commitment Scale of the Multigroup Ethnic Identity Measure-Revised (MEIM-R; Phinney & Ong, 2007). Sample items are: “I feel part of … culture” and “I feel a strong attachment towards … culture.” Responses were given on a forced-choice scale ranging from 1 (= *don’t agree at all*) to 4 (= *totally agree*). The internal consistency of the scales was α =. 93 (heritage culture) and α = 0.90 (host culture). The mean scores were 3.22 (*SD* = 0.80) for heritage identification and 2.69 (*SD* = 0.75) for host identification. Each construct was modeled as a latent variable in the multivariate structural equation modeling (SEM) analyses. Because the indicators of both constructs had strictly parallel wordings, the equivalence of the measurement models was assessed to see if mean values could also be compared. The measurement models showed excellent model fit and scalar equivalence (Kenny, 2020; see Table 2). The two factors were strictly orthogonal (*r*_*latent*_ = −0.011, *t* = −0.24, *p* = 0.81).

**Table 2:** Factor structure of cultural identification (unstandardized loadings, weighted data)

Indicators	Model 1: Parameters freely estimated		Model 2: Loadings equal	Model 3: Loadings and intercepts equal^a^
	Identification with heritage culture (HER)	Identification with host culture (HOS)		
I feel a strong attachment towards …	1.000	1.000	1.000	1.000
I feel part of …	1.025	1.109	1.054	1.054
I feel very comfortable …	1.005	1.022	1.012	1.012
I am very happy to be part of …	0.954	1.002	0.971	0.971
Parameter	25		22	18
Log likelihood	−6266.834		−6268.193	−6268.222
CFI	0.949		0.948	0.948
RMSEA	0.077		0.072	0.066
BIC	12699.434		1268.261	12655.796
Likelihood ratio test (Model 1 against Model 3/Model 2 against Model 3)	Chi^2^ = 3.56, *df* = 3, *p* = 0.31/ Chi^2^ = 0.054, *df* = 3, *p* = 0.997			

^a^Variances not significantly different (var_*HER*_ = 0.748, var_*HOS*_ = 0.6291; DIFF = 0.119, *t* = 1.535, *p* = 0.13)

### Academic, Social, and Psychological Adjustment

#### Academic Performance

Academic performance (AP) was indicated by grades in German, the first foreign language, mathematics, physics, and biology, as retrieved from the final grade 10 report card (α = 0.89). In Germany, these grades—and not performance tests—play a key role in determining students’ educational pathways and chances on the vocational training market.

#### School Attachment

School attachment (SATT) was assessed by eight items (α = 0.87) drawn from Salmela-Aro & Upadaya’s (2012) School Work Engagement Inventory and a scale on social and emotional well-being at school adopted from the Progress in International Reading Literacy Study (PIRLS; Martin et al., 2003; sample items: “I really enjoy some school activities”/“I feel comfortable at school”).

#### Self-esteem

Self-esteem (SE) was measured by four items (α = 0.85) from the KINDL questionnaire (Bullinger et al., 2008; sample items: “This past week I was satisfied with myself”; “This past week I was proud of myself”). The KINDL instrument represents a time-bound measure of self-esteem and is thus likely sensitive to state fluctuations. However, it captures the stable component of self-esteem similarly to Rosenberg’s Global Self-Esteem Scale (RSES; Rosenberg et al., 1995). In the present sample of adolescents aged 15 to 16, stability over one year was *r* = 0.50 without correction for attenuation. This value corresponds exactly to the age-appropriate uncorrected mean annual stability coefficient for self-esteem (RSES) found meta-analytically (Trzesniewski et al., 2003).

#### Life Satisfaction

Life satisfaction (LS) was assessed by an adapted 4-item version of the Satisfaction with Life Scale (SWLS; Diener et al., 1985; α = 0.91; sample item: “I am happy with my life right now”).

The scales tapping school attachment, self-esteem, and life satisfaction have all been established and validated in large-scale studies. All items have a forced-choice format, ranging from 1 (= *don’t agree at all*) to 4 (= *totally agree*).

The four adjustment dimensions were modeled as latent constructs with multiple indicators in the SEM analyses. A CFA with four factors showed good fit, with RSMEA = 0.051 and SRMR = 0.042 (Kenny, 2020). The latent correlations between the factors were *r*_*AP,SATT*_ = 0.23**, *r*_*AP,SE*_ = 0.09*, *r*_*AP,LS*_ = 0.03 ^ns^, *r*_*SATT,SE*_ = 0.13*, *r*_*SATT,LS*_ = 0.30**, *r*_*SE,LS*_ = 0.47**, indicating that the four factors represent distinct adjustment dimensions and that school attachment is clearly distinguishable from academic performance. Only the high correlation between self-esteem and life satisfaction indicates a potential collinearity problem.

All multivariate analyses controlled for a set of variables that have previously been found to covary with cultural orientation and/or dimensions of academic, social, and psychological adjustment and may act as confounders, as detailed in the following.

### Individual Characteristics and Family Background

#### Gender and Age

Students’ gender and age were retrieved from the school records.

#### Proficiency in German

Proficiency in German was measured by a reading literacy test from the OECD’s Program for International Student Assessment (PISA; OECD, 2019). The WLE scores of the IRT-scaled tests have a reliability of r_wle_ = 0.88. Proficiency in the heritage language was assessed by four self-report items: “How well can you write/read/speak/listen in the other language?” (α = 0.95). Responses were given on a 5-point scale from 0 (= *not at all*) to 4 (= *very well*). The mean score was 2.56 (*SD* = 1.46).

#### Socioeconomic Status (SES)

The family’s social status was measured using the International Socio-Economic Index of Occupational Status (ISEI); the highest value for either parent (HISEI), based on the occupations reported in the parent and student questionnaires, was entered in the analyses (Meraviglia et al., 2016).

#### Immigration Background

Immigrant background was operationalized as a dichotomous variable following the internationally accepted OECD (2019) standards (0 = both parents born in Germany, 1 = at least one parent born abroad). If these data were missing, data on language acquisition were used instead: Students who identified German as a second language were coded as having an immigrant background.

#### Immigrant Generation

Two immigrant generations were distinguished (1st = born abroad; 2nd = born in Germany).

#### Region of Origin

The assessment of cultural and ethnic background in this study warrants further explanation. In the U.S. Census, racial and ethnic diversity is assessed in terms of five races and one ethnicity, namely Hispanic or Latino (United States Census Bureau, 2021). By sharp contrast, in Europe—and Germany in particular—the term “race” has been consciously avoided since the Nazi regime and the Holocaust, on the assumption that its mere use can promote or even justify a biological essentialist understanding of phenotypical differences between people (see the experimental findings of Wilton et al., 2019). The same applies to the assessment of skin color. Instead, it has become standard practice to assess ethnic and cultural background in terms of the family’s country of origin. This approach is theoretically justifiable, as it generally also captures linguistic and legal communities that can well be the object of cultural identification. This holds especially when a religion has a privileged legal status, as is the case in Islamic countries and in some European countries (Fukuyama, 2018). For analytical purposes, individual countries had to be grouped into larger regions. This study took a pragmatic approach, constructing eight groups to reflect perceived cultural distance, privileging of religion, and dominant reasons for migration (unemployment, poverty, war and persecution), as detailed in the “Sample Description” section, and ranking them in order of perceived cultural distance following the findings of Schachner et al. (2014).

### School Characteristics

#### School track

School track was coded dichotomously (0 = ISS, 1 = academic-track *Gymnasium*).

#### Proportion of Immigrant Students

Representation of immigrant students at the school level was indexed by the percentage of 9th graders with immigrant background within a school. The mean score was *M* = 46.7 with a considerable standard deviation of *SD* = 27.5.

#### Ethnic Heterogeneity

The ethnic heterogeneity of the student body was measured by the Gini–Simpson Index. The Gini–Simpson Index indicates the probability that two students drawn at random from the same school have different ethnic backgrounds (calculated using $D = 1 - \mathop {\sum}\nolimits_{i = 1}^k {p_i^2}$, where *k* denotes the number of ethnicities in a 10th-grade cohort and *p* denotes the proportion of students belonging to the *i*th ethnicity). The theoretical range is from 0 to 1. The index varied significantly across schools (*M* = 0.46, *SD* = 0.23).

#### Multiculturalism Climate

Multiculturalism climate was measured in terms of the endorsement of multiculturalism beliefs by majority students in the grade cohort. The acculturation beliefs of majority peers are an indicator of the institutional acceptance of immigration. Normative multiculturalism beliefs were assessed by three items (α = 0.84) from the German version (Baumert et al., 2023) of the multiculturalism scale by Hahn et al. (2015; sample item: “I think that everyone benefits when many different cultures are represented in a school”). The items have a forced-choice format, ranging from 1 (= *don’t agree at all*) to 4 (= *totally agree*). The multiculturalism climate among majority group peers was assessed by aggregating individual scores at the level of the grade cohort of a school. The mean institutional multiculturalism climate score was positive and well above the neutral point, at 3.1, but the score showed considerable variation, mostly within the positive range (min. = 1.7, max. = 4.0, *SD* = 0.46). The intraclass correlation was ICC(1) = 0.22, indicating that 22% of the variance was between schools. With on average 10 raters per school, the ICC(2) as a measure of reliability was 0.74, a satisfactory score for group comparisons (Lüdtke et al., 2006). Indicators of faculty acculturation beliefs were not available.

### Missing Values and Multilevel Structure

Missing data due to dropout or nonresponse pose a widespread challenge in large-scale field studies. In this study, the missing rate depended on the data source: It was negligible for data retrieved from school records (gender, age) or report cards (grades), about 8% for achievement tests, up to 20% for questionnaire data, and about 40% for family background data, where written parental consent was needed. The Full Information Maximum Likelihood method (FIML), which considers the covariance matrix for all available data to estimate parameters (Enders, 2010), was therefore applied. All variables used in the full multivariate models were also included as auxiliary variables in all other models to ensure efficient parameter estimates (Graham, 2003).

Due to the hierarchical design of the BERLIN study, individuals are nested within schools. Depending on the intraclass correlation, this can result in the underestimation of standard errors. Robust standard errors that take dependencies into account are therefore reported.

### Statistical Analyses

The focus of this study is to analyze the relationship between cultural orientation and a variety of adjustment dimensions, taking into account additive and multiplicative effects. Person-centered approaches to conceptualizing cultural identity are less appropriate for these purposes. The present analyses therefore follow the recommendations of Arends‐Tóth and van de Vijver (2007) and Rudmin (2009) on a bilinear dimensional approach. SEM analyses with latent modeling of cultural identification and adjustment were the first choice for estimating the interrelationships. Addressing the interplay of cultural identification *within* specific dimensions of adjustment—irrespective of the covariation of the dimensions and their shared variance—suggests running SEM analyses separately by adjustment dimension, particularly when there is potential multicollinearity among adjustment dimensions (see Measures). Multiple testing generally increases the probability of an inflated alpha error. However, when comparisons are few and planned, correcting for multiple comparisons is not recommended, as in this case the adjustments might be too conservative (Keppel & Wickens, 2004; Streiner & Norman, 2011). In the present study, the following approach was taken: All analyses with outcome variables that were only weakly correlated (*r* < 0.25: academic performance, school attachment, and self-esteem) were performed concurrently to avoid multiple testing. For life satisfaction, which was substantially correlated with school attachment (*r* = 0.30) and self-esteem (*r* = 0.47), analyses were conducted separately without correction for multiple testing.

Each SEM analysis involved two steps, consistent with the theoretical model presented in Fig. 1. First, a core model specifying only the main effects of cultural identification and their interaction was fitted. Second, covariates and their potential moderator function were entered in the model. To control for multicollinearity of the covariates, variance inflation factors were computed using the method proposed by Marcoulides and Raykov (2019). For reasons of statistical parsimony, institutional characteristics (school type, proportion of students with immigrant background, and school diversity climate) were modeled on the individual level with robust standard errors, as no cross-level interactions were specified. In all analyses, correlations between exogenous variables were freely estimated. All multivariate analyses were performed with Mplus 8.6 (Muthén & Muthén, 1998–2017).

Because this study is based on observational data, it is subject to the problems of unobserved heterogeneity, selective intake, and possibly omitted variable bias. Despite careful control of potential confounders, caution is warranted in drawing any causal inferences.

## Results

### Sample Description

Table 3 describes the sample of *N* = 842 students with immigrant background. At Wave 1, their mean age (15.3 years) did not differ from that of the majority group (15.2 years). While 59.7% reported that both parents were born abroad, 40.3% had one German-born parent. Most students with immigrant background belong to the second immigrant generation, having been born in Germany (78.0%), and grew up bilingually (80.5%). Of those born elsewhere (22.0%), most moved to Germany before starting school. Nevertheless, adolescents with immigrant background did not reach the average German language proficiency of their native peers by the end of compulsory schooling. The difference was on average *d* = −0.52; it was largest for adolescents of the first immigrant generation with *d* = −0.65, but still *d* = −0.31 in the second generation. The mean HISEI score for families with immigrant background was 51.7, relative to 60.0 for those without—the significant difference of around half a standard deviation indicates that immigration is associated with lower social status in Germany.

**Table 3:** Sample description, language proficiency, and cultural identification by individual characteristics (means, standard deviations, paired sample test for immigrant background, figures for majority students in brackets)^a^

Individual characteristics	Distribution parameter	Command of German^b^		Command of heritage language^c^		Cultural identification				Paired sample *t* test for cultural identification^d^		
						Heritage		Host				
	% or *M*/*SD*	*M*	*SD*	*M*	*SD*	*M*	*SD*	*M*	*SD*	*t* (*df*)	*p*	*d*
Total		−0.26 [0.26]	1.00 [0.94]	0.00	1.00	3.22	0.80	2.69 [2.91]	0.75 [0.74]	13.84 (700)	<0.01	0.74
Age (in years, May 2014; *M*/*SD*)	15.3/0.68 [15.2/0.59]	–	–	–	–	–	–	–	–	–	–	–
SES (HISEI; *M*/*SD*)	51.7/20.3 [60.0/18.7]	–	–	–	–	–	–	–	–	–	–	–
Gender												
Female	50.1 [46.8]	−0.15	0.97	0.08	0.97	3.30	0.77	2.66	0.72	10.47 (339)	<0.01	0.91
Male	49.9 [53.2]	−0.38	1.02	−0.08	1.02	3.14	0.82	2.72	0.78	7.935 (342)	<0.01	0.59
Immigration background												
One parent	40.3	0.08	0.95	−0.31	1.16	3.01	0.88	2.93	0.71	1.72 (172)	0.09	0.17
Both parents	59.7	−0.16	0.88	0.21	0.85	3.34	0.75	2.66	0.68	11.39 (302)	<0.01	0.92
Immigrant generation												
First	22.0	−0.39	0.99	0.24	0.78	3.22	0.77	2.58	0.70	6.90 (124)	<0.01	0.88
Second	78.0	−0.05	0.92	0.03	0.92	3.21	0.82	2.79	0.72	8.24 (406)	<0.01	0.60
Language acquisition												
Simultaneous bilingual	44.3	−0.20	0.95	0.41	0.56	3.29	0.74	2.75	0.77	9.44 (320)	<0.01	0.53
Sequential bilingual	36.2	−0.41	1.02	0.49	0.47	3.34	0.75	2.56	0.71	12.43 (272)	<0.01	0.78
German monolingual	19.5	−0.03	1.03	−1.75	0.00	2.61	0.88	2.96	0.66	−2.14 (76)	0.04	0.24
Country/region of origin^e^												
Former USSR and Poland	16.4	−0.02	0.89	0.07	0.94	3.09	0.76	2.81	0.67	3.17 (117)	<0.01	0.40
USSR	9.0	0.04	0.80	0.19	0.80	3.11	0.72	2.84	0.673	2.17 (63)	0.03	0.42
Poland	7.4	−0.09	0.99	−0.09	1.09	3.04	0.81	2.730	0.693	2.22 (53)	0.03	0.29
Former Yugoslavia	8.6	−0.37	0.90	0.10	0.93	3.41	0.80	2.56	0.841	5.45 (58)	<0.01	1.04
Other European country^f^	8.8	0.05	1.01	−0.54	1.17	2.99	0.84	2.70	0.863	2.16 (57)	0.04	0.34
Turkey	30.8	−0.34	1.00	0.21	0.92	3.45	0.72	2.59	0.76	12.48 (232)	<0.01	1.20
Middle East/North Africa^g^	15.8	−0.53	1.02	−0.05	0.90	3.23	0.81	2.73	0.72	5.149 (118)	<0.01	0.71
Asia	8.1	0.03	0.86	−0.06	0.98	2.95	0.82	2.75	0.663	1.45 (64)	0.15	0.27
Sub-Saharan Africa	2.0	−0.19	0.83	−1.69	0.94	2.67	0.89	2.60	0.413	0.375 (16)	0.71	0.15
Other non-European country^h^	9.4	0.04	1.09	−0.39	1.20	3.32	0.81	2.53	0.84	3.82 (58)	<0.01	0.96
South or Central America	1.4	0.03	0.87	−0.27	1.19	3.24	0.65	2.52	0.84	2.21 (10)	0.05	1.12
North America, Australia, or New Zealand	0.4	–	–	–	–	–	–	–	–	–	–	–
Unspecified	7.6	0.04	1.13	−0.41	1.20	3.147	0.783	2.622	0.762	3.15 (47)	<0.01	0.68

^a^Weighted data

^b^Standardized in the total sample

^c^Standardized in the sample of students with immigrant background

^d^The paired sample *t* test is used to capture intraindividual differences between a given student’s heritage and host identification

^e^Ordered according to cultural distance as perceived by secondary students in Germany (Schachner et al., 2014, Table 2)

^f^Predominantly southern and south-eastern European countries

^g^Including Afghanistan, Iran, and Maghreb countries

^h^Not ranked according to perceived cultural distance

The students’ families came from a variety of countries and cultures. Schachner et al. (2014) have shown that 12-year-olds asked to gauge the cultural distance of various immigrant groups from the majority culture already show relatively high levels of agreement (for 15-year-olds, see Schachner et al., 2017; for adults, see Heath & Richards, 2020). Building on the findings of Schachner et al. (2014), in this study the countries of origin were grouped as follows: (1) Most immigrants from the former Soviet Union (USSR) and Poland are *Spätaussiedler*—people with German ancestry who moved to Germany after the collapse of the Eastern bloc and were generally granted German citizenship. This group accounts for 16.4% of students with immigrant background in the present sample. In the SEM analyses, they constitute the reference group of those with the smallest cultural distance from the host population. (2) Another 8.6% hail from countries of the former Yugoslavia; most of their families fled to Germany during the civil war, and (3) 8.8% from other (primarily southern) European countries; their families came to Germany in search of work. (4) The largest group (30.8%) is from families with Turkish heritage, most of whom came to Germany as “guest workers” in the 1960s and 1970s, and more than 90% of whom are Muslim. (5) Another primarily Muslim group consists of students with Middle Eastern or North African heritage (15.8%). Most immigrants from these countries fled war or persecution. (6) Students with Asian heritage make up 8.1% of the sample. Their parents came to the former German Democratic Republic (GDR) as part of socialist exchange programs or in search of work. (7) A small group of students (2.0%) come from families who moved from sub-Saharan African countries to escape poverty or civil war. (8) For two small groups originating in (a) South or Central America (1.4%) and (b) North America, Australia, or New Zealand (0.4%), as well as (c) a residual group from unspecified non-European countries (7.6%), no information on perceived cultural distance was available. According to the findings of the European Social Survey, groups 4 and 5—mostly Muslims from Turkey, the Middle East and North Africa—are the least accepted by the majority society (Heath & Richards, 2020).

### Patterns of Cultural Identification

Table 3 also presents identification with the heritage and host culture by individual background characteristics. The mean scores for both were above the neutral point of 2.5 (range: 1–4), but heritage scores were notably higher (mean difference: *d* = 0.74). This result is in line with international findings from the ICSEY study (Phinney et al., 2006). As expected, adolescents of the second immigrant generation reported stronger identification with the host culture than did those of the first generation (*F*_(1,810)_ = 4.82, *p* = 0.02; *d* = 0.30), but the two did not differ significantly with respect to heritage identification (*F*_(1,726)_ = 0.03, *p* = 0.85). Students with one parent born abroad identified more strongly with the host culture (*F*_(1,526)_ = 8.76, *p* < 0.01; *d* = 0.39) and less strongly with the heritage culture (*F*_(1,472)_ = 11.75, *p* < 0.01; *d* = −0.41) than those whose parents were both born abroad. Overall, identification with the heritage and host culture were orthogonal (*r* = −0.011 ^ns^).

Systematic differences in immigrant groups’ cultural identification were expected (Hypothesis 1a), with host identification decreasing and heritage identification increasing as cultural distance grows. This hypothesis was tested by means of two ANOVAs with cultural identification as the dependent variable and region of origin as the independent variable. Contrary to expectations, no significant differences emerged for host identification (*F*_(7,752)_ = 1.67, *p* = 0.11). Post hoc comparisons between individual groups confirmed this pattern. The overall test for heritage identification was significant (*F*_(7,692)_ = 4.10, *p* < 0.01). Post hoc comparisons with Bonferroni correction showed that young people with Turkish heritage, most of whom were Muslim, were more committed to the heritage culture than all other groups (with the exception of those with Yugoslavian heritage). The intraindividual difference between the two cultural orientations was also most pronounced in this group, at *d* = 1.20. In contrast, no post hoc comparison was significant for adolescents of Middle Eastern heritage, most of whom were also Muslim. Contrary to expectations, the heritage scores of students with Yugoslavian heritage were significantly higher than those of students with Asian or sub-Saharan African heritage, although the latter groups are perceived to be more culturally distant. No specific identification pattern could be identified for People of Color or young people whose families had experienced war, persecution, and flight. In sum, the observed differences are inconsistent with the pattern predicted in Hypothesis 1a.

A typological approach, as proposed by Berry et al. (1989), was used to examine whether the likelihood of dual identification differed by origin group. All other analyses took a dimensional approach, as explained in the Methods section. The two cultural identification scales were dichotomized at their neutral point (2.5) into “committed” and “noncommitted” and crossed to produce the Berry et al. (1989) typology of cultural orientations. Within this typology, 43.4% of 15–17-year-olds with immigrant background identified with both cultures, 34.5% with only the heritage culture, 10.7% with only the host culture, and 11.3% percent with neither, indicating that they were distanced from both cultures. The likelihood of dual identification did not differ significantly across immigrant groups (*F*_(7,627)_ = 0.31, *p* = 0.95).

Dichotomizing the two scales at the neutrality point has met with criticism, as young people who were indecisive would be forced into one of the four categories (e.g., Schwartz & Zamboanga, 2008). A sensitivity analysis was therefore performed with five categories, in which individuals whose scores fell between agreement (score 3) and disagreement (score 2) were assigned to a separate category of “undecided.” Within this typology, 30.2% of 15–17-year-olds with immigrant background identified with both cultures, 40.6% with only the heritage culture, 12.5% with only the host culture, and 4.4% percent with neither. Another 12.4% were undecided and possibly still in an exploration phase of their cultural identity (Meeus et al., 2010). Again, the likelihood of dual identification did not differ significantly across immigrant groups (*F*_(7,627)_ = 0.41, *p* = 0.90).

### Cultural Identification and Academic, Social, and Psychological Adjustment: SEM Analyses

All SEM analyses comprised two steps. First, a baseline model specifying only the main effects of cultural identification and their interaction was fitted. Second, the analyses controlled for stable personal characteristics (gender, SES, immigrant generation), region of origin, and institutional context, including school track, proportion of students with immigrant background, ethnic heterogeneity, and the multiculturalism climate of the school. Religion was not specified due to collinearity with region of origin. Whether to control for proficiency in the majority language and the language of origin (see Table 3) is a theoretical question. Mastery of each language develops in interplay with the corresponding cultural orientation, as Hochman and Davidov (2014) showed in an autoregressive cross-lagged model. Both language proficiency and cultural orientation are indicators of cultural commitment. Consequently, statistically controlling for language proficiency would partial out theoretically relevant parts of the variance in cultural orientations and potentially underestimate the associations between cultural orientation and adjustment. Therefore, proficiency in German and the heritage language was not specified in the SEM models. Additional sensitivity analyses examined interactions between immigrant generation and cultural identification, and between region of origin and cultural identification. Overall, only two significant interactions emerged, indicating that most findings seemed to be relatively robust across immigrant generation and culture of origin. The significant interactions are discussed below. Variance inflation factors and their confidence intervals were computed for all covariates. In line with the weak correlations reported in Table 4, the upper limits of all confidence intervals stayed below the critical limit (5.0) for multicollinearity (Marcoulides & Raykov, 2019). Analyses with academic performance, school attachment, and self-esteem were conducted concurrently. Analyses for life satisfaction were run separately (see Methods). Table 5 summarizes the results of the SEM analyses on the relationship between cultural identity and dimensions of adjustment. All coefficients of metric predictors and interactions are fully standardized; coefficients of dichotomous predictors are *y*-standardized. This means that if the value of a predictor shifts by one standard deviation or one category, the outcome variable follows with *β* standard deviations. Sensitivity analyses comparing separate and concurrent estimations (see Appendix, Table A1) justify the chosen approach. They show that estimations of parameters and standard errors differed only marginally—except when life satisfaction was included.

**Table 4:** Bivariate latent correlations between adjustment, cultural identification, and covariates

	1	2	3	4	5	6	7	8	9	10	11	12	13
Academic performance (1)	1.0												
School attachment (2)	0.23	1.0											
Self-esteem (3)	−0.08	0.13	1.0										
Life satisfaction (4)	0.04	0.31	0.47	1.0									
Heritage identification (5)	−0.12	0.12	0.13	0.12	1.0								
Host identification (6)	0.16	0.25	−0.03	0.20	−0.05	1.0							
Gender (7)	0.17	−0.03	−0.22	−0.17	0.11	−0.02	1.0						
SES (8)	0.10	−0.02	−0.29	−0.15	−0.08	0.06	0.00	1.0					
2nd generation (9)	0.16	0.12	−0.04	0.06	−0.03	0.14	0.06	0.09	1.0				
Multiculturalism climate in the grade cohort (10)	0.22	0.03	−0.10	−0.03	0.08	0.00	0.09	0.02	0.12	1.0			
Proportion students with immigrant background in the grade cohort (11)	−0.14	0.02	0.11	−0.01	0.14	−0.08	0.04	−0.22	0.03	0.10	1.0		
Ethnic heterogeneity (12)	−0.07	0.02	0.02	−0.08	0.09	−0.12	0.11	−0.14	0.04	0.10	0.67	1.0	
School track (*Gymnasium*) (13)	0.35	0.11	−0.14	0.01	−0.11	0.06	0.15	0.10	0.16	0.52	0.02	0.04	1.0
Region of origin (Reference: former USSR and Poland)													
Former Yugoslavia	−0.08	−0.05	−0.02	−0.00	0.09	−0.05	0.02	−0.13	0.00	−0.02	0.06	0.06	−0.03
Other European countries	0.02	−0.09	−0.01	−0.04	−0.10	0.03	0.02	0.12	0.00	0.06	−0.11	−0.07	−0.04
Turkey	−0.12	0.04	0.05	0.04	0.19	−0.09	−0.01	−0.1	0.21	0.00	0.21	0.11	0.03
Middle East/North Africa	−0.06	0.01	0.12	0.11	−0.01	0.05	−0.01	0.02	−0.02	0.03	0.08	0.06	−0.08
Asia	0.16	0.05	−0.09	−0.10	−0.10	0.01	0.01	0.03	0.01	0.03	−0.06	−0.06	0.07
Sub-Saharan Africa	0.04	−0.04	−0.02	−0.06	−0.10	−0.03	0.03	−0.03	0.03	−0.04	0.01	0.01	0.03

**Table 5:** SEM analyses: regression of academic performance, school attachment, self-esteem, and life satisfaction on cultural identification and covariates (parameters for academic performance, school attachment, and self-esteem concurrently estimated, standardized regression coefficients, robust standard errors in parentheses, *N* = 833)

Predictors	Academic performance^a^		School attachment^b^		Self-esteem^c^		Life satisfaction^d^		
	Model 1	Model 2	Model 1	Model 2	Model 1	Model 2	Model 1a 1st generation *N* = 137^e^	Model 1b 2nd generation *N* = 450^e^	Model 2 2nd generation *N* = 450^e^
*Cultural identification*									
With host culture (HOS)^f^	0.187** (0.050)	0.131** (0.045)	0.264** (0.057)	0.334** (0.067)	−0.032 (0.063)	0.008 (0.056)	0.281** (0.104)	0.171** (0.068)	0.140* (0.069)
With heritage culture (HER)^f^	−0.138** (0.056)	−0.129** (0.048)	0.127* (0.060)	0.133* (0.053)	0.117* (0.053)	0.158** (0.046)	−0.034 (0.116)	0.204** (0.065)	0.161** (0.052)
Interaction HOS × HER	−0.167** (0.056)	−0.076^+^ (0.045)	−0.061 (0.063)	–	−0.034 (0.077)	–	0.010 (0.111)	−0.265** (0.049)	−0.278** (0.046)
Interaction HOS × Turkish origin				−0.121* (0.056)					
*Individual characteristics*									
Gender (female)^g^		0.290** (0.081)		−0.107 (0.081)		−0.428** (0.077)			−0.291** (0.099)
SES (HISEI)		0.231 (0.110)		−0.066 (0.073)		−0.255** (0.075)			−0.191** (0.066)
Immigrant generation (second)^g^		0.174 (0.176)		0.205 (0.149)		0.127 (0.156)			
*Region of origin (Ref.: former USSR and Poland)*									
Former Yugoslavia^g^		−0.139 (0.211)		−0.256 (0.220)		−0.235^+^ (0.167)			−0.185 (0.323)
Other European country^g^		−0.017 (0.208)		−0.339 (0.220)		−0.032 (0.166)			−0.029 (0.160)
Turkey^g^		−0.027 (0.188)		−0.117 (0.161)		−0.119 (0.147)			−0.159 (0.203)
Middle East^g^		−0.085 (0.169)		−0.155 (0.121)		0.251 (0.178)			0.122 (0.176)
Asia^g^		0.422* (0.218)		0.0814 (0.181)		−0.217 (0.157)			−0.402^+^ (0.220)
Sub-Saharan Africa^g^		0.172 (0.352)		−0.237 (0.260)		0.054 (0.289)			−0.356 (0.426)
*Institutional characteristics*									
School track (*Gymnasium*)^g^		0.455** (0.123)		0.198 (0.131)		−0.0751(0.072)			0.197 (0.118)
Proportion students with immigrant background in grade cohort (%)		−0.113* (0.059)		0.001 (0.068)		0.455* (0.217)			0.177* (0.076)
Ethnic heterogeneity (Gini–Simpson Index)		0.035 (0.055)		−0.038 (0.062)		−0.074 (0.057)			−0.218** (0.078)
Multiculturalism climate (MC)^h^		0.128* (0.060)		−0.051 (0.062)		−0.107** (0.041)			−0.003 (0.069)
*R*^*2*^	0.083* (0.029)	0.257** (0.038)	0.087** (0.026)	0.126** (0.034)	0.016 (0.015)	0.178** (0.044)	0.079 (0.059)	0.134** (0.036)	0.260** (0.060)

^a^Latent construct; measurement model: final grades in German, the first foreign language, math, physics, and biology at the end of grade 10

^b^Latent construct; measured with eight indicators (see Methods)

^c^Latent construct; measured with four indicators (see Methods)

^d^Latent construct; measured with four indicators (see Methods)

^e^*N*_total_ = 587 due to missing values on generation.

^f^Latent construct; measured with four indicators (see Methods and Table 2)

^g^Regression coefficients *y*-standardized

^h^Regression HOS on MC: *β* = 0.017 (0.034) and HER on MC: *β* = 0.088 (0.059)

**p ≤ 0.0.01, *p ≤ 0.05, ^+^*p* ≤ 0.10

#### Cultural Identification and Academic Performance

**Fig. 2:**
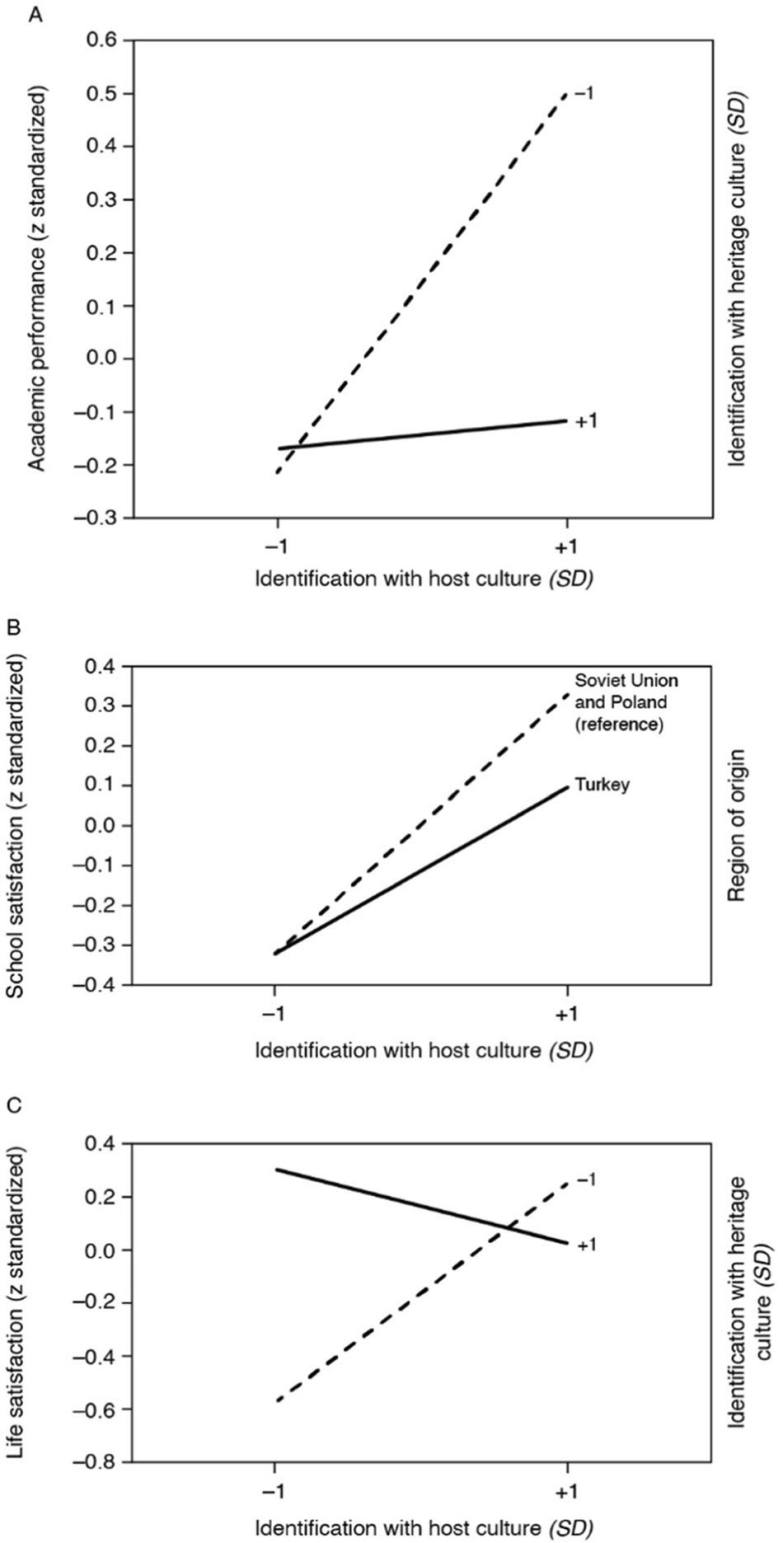
Visualization of Interactions for (**A**) Academic Performance, (**B**) School Satisfaction, and (**C**) Life Satisfaction

Findings for academic performance, measured in terms of grades in the final grade 10 report card, are presented in the first block of Table 5. In Model 1, identification with the heritage and host culture and their interaction were entered as predictors. The results show two opposing main effects—a positive effect for host identification (*β*_*HOS*_ = 0.19, *t* = 3.78, *p* < 0.01) and a negative effect for heritage identification (*β*_*HER*_ = − 0.14, *t* = −2.46, *p* < *0*.01)—and a significant negative interaction effect (*β*_HOS*HER_ = −0.17, *t* = −2.97, *p* < 0.01). The two opposing main effects are in line with Hypothesis 2a; no directional hypothesis was formulated for the interaction effect. Figure 2a visualizes these findings: host identification served as an individual resource for academic performance. Strong heritage identification reduced the positive effects of strong host identification, but was not associated with a further drop in performance when host identification was low. A sensitivity analysis comparing separate and concurrent estimations revealed only marginal differences (see Appendix, Table A1).

Model 2, which controlled for stable background variables and the institutional context, showed the same pattern of results but with weaker effects. The opposing main effects were now of the same size (*β*_*HOS*_ = 0.13, *t* = 2.90, *p* < 0.01; *β*_*HER*_ = −0.13, *t* = −2.68, *p* < 0.01), and the negative interaction was only marginally significant. Rivas-Drake et al. (2014) hypothesized that heritage orientation mediated by self-esteem might have a positive effect on academic performance—if this were the case, the heritage effect would be negatively biased in the model. However, the coefficient changed only marginally with control for self-esteem (*β*_*HER*_ = −0.12**). Overall, host identification appeared to constitute an academic resource and heritage identification a potential risk factor—a finding that contradicts the Additive Resource Model of biculturalism. No interactions emerged between cultural identification and immigrant generation or region of origin.

Inspection of the covariates shows that girls with immigrant background were much better able to cope with the academic demands of school than were boys with immigrant background (*β*_*girls*_ = 0.29, *t* = 3.56, *p* < 0.01). Consistent with previous findings (OECD, 2019), SES was positively correlated with academic performance (*β*_*SES*_ = 0.23, *t* = 2.10, *p* = 0.04). Second-generation students performed better academically than their first-generation peers (see Tables 1 and 3). However, with control for cultural identity, this difference was no longer significant (*β*_*2ndGEN*_ = 0.17, *t* = 0.99, *p* = 0.32). These findings stand in contrast to the “immigrant paradox” observed in the United States, where academic adjustment tends to be lower in the second generation than in the first (García Coll & Marks, 2012), but are consistent with findings from other European countries (Dimitrova et al., 2017). In terms of region of origin, students with Asian heritage were by far the most successful group, outperforming the other immigrant groups by almost half a standard deviation (*β*_*Asian*_ = 0.42, *t* = 1.193, *p* = 0.05); the other groups did not differ significantly from the reference group. There was no (negative) interaction between Asian origin and heritage identification that weakened the negative effect of heritage orientation, indicating that identification with Asian culture might convey family-driven aspirations for academic excellence.

Controlling for institutional characteristics of the school attended revealed a differentiated picture. As expected, students in the selective academic track (*Gymnasium*) performed significantly better (*β*_*track*_ = 0.46, *t* = 3.70, *p* < 0.01) than their peers in ISS. As the proportion of students with immigrant background in the grade cohort increased, the academic performance of students with immigrant background tended to decrease (*β*_*%imm*_ = −0.11, *t* = −1.93, *p* = 0.053). In contrast, the ethnic heterogeneity of the student body was not related to academic performance (*β*_*HET*_ = 0.04, *t* = 0.64, *p* = 0.53). Of particular theoretical interest was the extent to which the multiculturalism beliefs of majority peers were associated with the cultural orientation and academic performance of students with immigrant background. The results were remarkably clear: A school’s multiculturalism climate (MC) was positively related to academic performance (*β*_*MC*_ = 0.13, *t* = 2.13, *p* = 0.03). The coefficients of the regression of cultural identification on MC were not significant (*β*_*HOS_MC*_ = 0.02, *t* = 0.49, *p* = 0.62; *β*_*HER_MC*_ = 0.09, *t* = 1.51, *p* = 0.13). Thus, Hypothesis 1b, which predicted that the cultural orientations of students with immigrant background would depend on the diversity beliefs of majority peers, was not confirmed.

#### Cultural Identification and School Attachment

The second block of Table 5 presents findings for school attachment. School attachment was conceptualized as motivational, social, and emotional engagement in curricular and extracurricular school activities (see Measures) and clearly distinguished from academic performance (*r*_*AP,SATT*_ = 0.23**). In Model 1, both host and heritage identifications seem to constitute individual resources for school attachment, with host identification (*β*_*HOS*_ = 0.26, *t* = 4.67, *p* < 0.01) having a stronger effect than heritage identification (*β*_*HER*_ = 0.13, *t* = 2.13, *p* = 0.03). The interaction between the two orientations was not significant (*β*_HOS*HER_ = − 0.06, *t* = −0.96, *p* = 0.34). These findings are in line with Hypothesis 2b and the Additive Resource Model of biculturalism. A sensitivity analysis comparing separate and concurrent estimations revealed only marginal differences (see Appendix, Table A1).

Model 2 controlled for covariates and relevant interactions. No interactions were found between cultural orientations and immigrant generation; however, a negative interaction emerged between host identification and Turkish heritage (*β*_HOS*Turk_ = −0.12, *t* = −2.16, *p* = 0.03), indicating that the positive relationship between host identification and school attachment was attenuated in this group. Figure 2b illustrates the moderation effect. Overall, there was little change when covariates were included in Model 2. Surprisingly, school attachment seemed to be largely independent of both individual background and institutional characteristics, including multiculturalism climate. Although the regression coefficients suggest that second-generation students and those with families from the former Soviet Union and Poland were somewhat better integrated in school, the test power was not sufficient to statistically confirm the differences.

#### Cultural Identification and Psychological Adjustment

The third block of Table 5 presents the findings for self-esteem. In line with Hypothesis 2c, a moderate positive main effect emerged for heritage identification in the baseline model (Model 1: *β*_*HER*_ = 0.12, *t* = 2.23, *p* = 0.01), which increased when potential confounders were controlled (Model 2: *β*_*HER*_ = 0.16, *t* = 3.46, *p* < 0.01). Contrary to Hypothesis 2c, however, host identification was not positively associated with self-esteem (*β*_*HOS*_ = − 0.03, *t* = −0.44, *p* = 0.66). Thus, the findings did not support the Additive Resource Model of biculturalism. Interactions between cultural identification and immigrant generation or region of origin were not found. A sensitivity analysis comparing separate and concurrent estimations revealed only marginal differences when life satisfaction was not included; including life satisfaction in the concurrent estimation modified the meaning of self-esteem and accordingly the parameter estimates (see Appendix, Table A1).

Model 2 corroborates previous findings (Piccinelli & Wilkinson, 2000) that girls between 15 and 17 years of age experience much more self-doubt than their male peers, independent of region of origin (interactions were not significant); the effect size was substantial (*β*_*girls*_ = −0.43, *t* = −5.57, *p* < 0.01). Adolescents from more socially privileged families also reported lower self-esteem (*β*_*SES*_ = −0.26, *t* = −3.41, *p* < 0.01). The self-esteem of first- and second-generation students did not differ significantly. Differences between immigrant groups were not significant. With respect to institutional characteristics, self-esteem appeared to stabilize with an increasing proportion of students with immigrant background in the grade cohort (*β*_*%*_ = 0.46, *t* = 2.09, *p* = 0.04), but was independent of the ethnic heterogeneity of the student body (*β*_*HET*_ = −0.07, *t* = −1.29, *p* = 0.20). Unexpectedly, endorsement of multiculturalism among majority peers was negatively related to the self-esteem of students with immigrant background (*β*_*MC*_ = −0.11, *t* = −2.64, *p* < 0.01). This finding contradicts Hypothesis 1b, but is consistent with the experimental findings of Wilton et al. (2019), who reported increased self-stereotyping of minorities when categorical differences were accentuated by endorsement of multiculturalism beliefs (see also Rios, 2022).

The fourth block of Table 5 presents the findings for life satisfaction. Because a significant negative interaction emerged between heritage identification and immigrant generation, all analyses were conducted separately by immigrant generation. For the first generation, Model 1a showed a significant positive effect for host identification (*β*_*HOS*_ = 0.28, *t* = 2.70, *p* < 0.01), which was no longer significant with control for covariates (*β*_*HOS*_ = 0.12, *t* = 0.74, *p* = 0.46; not reported in Table 5). For the second generation, Model 1b showed positive main effects for host identification (*β*_*HOS*_ = 0.17, *t* = 2.51, *p* = 0.01) and heritage identification (*β*_*HER*_ = 0.20, *t* = 3.14, *p* < 0.01), as well as a negative interaction (*β*_HOS*HER_ = − 0.27, *t* = −5.36, *p* < 0.01). Figure 2c illustrates this finding. Identifying with either the heritage culture or the host culture represented a sufficient and mutually substitutable resource for life satisfaction. Biculturalism did not offer an additional gain, but rather implied a small loss. Dual cultural distance, however, appeared to be a cumulative risk factor, consistent with Berry’s marginalization hypothesis (Berry et al., 2006). Comparison with the findings for the first generation shows that host identification and especially heritage identification became mutually substitutable resources only in the second generation. A sensitivity analysis across both generations comparing separate and concurrent estimation revealed that the concurrent estimates were not completely robust due to collinearity between self-esteem and life satisfaction (see Appendix, Table A1).

Including covariates in Model 2 showed the following picture. Analogously to the findings for self-esteem, girls and adolescents from more socially privileged families reported lower life satisfaction. The effect size for gender was substantial (*β*_*girls*_ = −0.29 *t* = −2.94, *p* < 0.01); that for SES was moderate (*β*_*SES*_ = − 0.19, *t* = −2.92, *p* < 0.01). Differences between origin groups varied nonsystematically and were marginally significant only in the case of lower life satisfaction among adolescents of Asian origin (*β*_*Asian*_ = −0.40, *t* = −1.83, *p* = 0.07). In terms of the institutional context of the school attended, life satisfaction of students with immigrant background increased with the proportion of peers with immigrant background in the grade cohort (*β*_*%*_ = 0.18, *t* = 2.32 *p* = 0.02), but decreased as ethnic heterogeneity in the grade cohort increased (*β*_*HET*_ = −0.22, *t* = 2.79, *p* < 0.01). Life satisfaction was not related to the multiculturalism beliefs of majority peers (*β*_*MC*_ = −0.003, *t* = −0.05, *p* = 0.96).

## Discussion

How the cultural identity formation of ethnic minority adolescents relates to their academic, social, and psychological adjustment is an important and as yet unresolved research question. This applies in particular to host societies in which increasing ethnic and cultural diversity on the individual level collides with institutional conservativism and xenophobia in the majority population, and where not all immigrant groups are equally welcome. How does the societal and institutional context affect the identity development of immigrant youth? This study attempts to fill this research gap by examining the relationship between cultural identity formation and perceived distance from the majority culture and by considering multiple dimensions of adjustment simultaneously.

The study’s findings on patterns of cultural identification among adolescents with immigrant background in Berlin were remarkably clear: Identification with the heritage and the host culture were orthogonal, indicating that—despite the polarized public debate on immigration (Holloway et al., 2021)—all cultural identifications were in principle open to adolescents with immigrant background. Orthogonality of the two orientations is uncommon in early and mid-adolescence. Most previous studies have reported negative correlations of medium size (e.g., Schwartz et al., 2007; though Karataş et al., 2023, reported mixed results). In Germany, a negative correlation (*r* = −0.35) has also been observed in a large sample representative for the 15-year-old population; the correlation became positive at age 18 years (*r* = 0.08) and increased to *r* = 0.17 at age 24 years (NEPS; https://www.neps-data.de). Orthogonality at age 15 years seems to be specific to metropolitan areas with a large and diverse immigrant population (Schwartz & Unger, 2010). On average, young people were committed to both cultures, with heritage orientation being more pronounced (*M*_*HER*_ = 3.22; *M*_*HOS*_ = 2.69, *d* = 0.74). Although commitment to the heritage culture differed by region of origin, the differences were unsystematic and inconsistent with Hypothesis 1a, which predicted that heritage identification would increase with growing cultural distance from the majority culture. These findings contradict the hypothesis of “reactive” ethnic identity formation (Rumbaut, 2008; Schwartz et al., 2018). Remarkably, heritage identification was more pronounced in young people whose families had immigrated from countries of the former Soviet Union or Poland and who were privileged naturalized citizens of Germany. This indicates that the heritage culture can have an enduring impact on identity formation in adolescence, even if the family is legally fully integrated into the host society. From a typological perspective, the most important identification pattern was biculturalism: Depending on the classification approach, between 30% and 42% of young people with immigrant background identified with both the heritage and the host culture. The likelihood of dual identification was independent of the culture of origin.

This pattern of results corroborates the few previous findings (Schachner et al., 2014, 2017; Yağmur & van de Vijver, 2012) and suggests that differences in the acceptance of immigrant groups among the adult majority population (Heath & Richards, 2020) are not directly related to the development of cultural identity in adolescents. This is an important finding, which indicates that potential differences in adjustment between adolescents of different cultural origins are not mediated by cultural identification. Surprisingly, the normative diversity beliefs shared by majority group peers in schools—an important feature of adolescents’ proximal developmental environment (Suárez-Orozco et al., 2018)—did not seem to be associated with cultural identity formation either (Hypothesis 1b). One explanation could be that cultural diversity is generally accepted among the younger generation (see Measures; Baumert et al., 2023), making schools—despite considerable variation in the diversity climate (*ICC* = 0.22)—a developmental environment that may serve as a buffer against societal rejection. Alternative explanations are that diversity-friendly attitudes only reflect behavior to a limited extent and that discrimination and microaggressions also vary between schools, or that the diversity climate of a school is determined more by the acculturation beliefs of the faculty than by those of the student body.

The present findings on the relationships between cultural identification and dimensions of adjustment paint a differentiated and highly domain-specific picture. In the context of academic adjustment, an immigration background is often regarded as a risk factor for long-term sociostructural integration due to the family’s cultural distance to the school system of the host society and the typically lower proficiency in the language of instruction (see Table 3). This risk is assumed to be mitigated by strong identification with the host culture and greater investment in language learning (Hochman & Davidov, 2014)—and potentially exacerbated by strong identification with the heritage culture (Schotte et al., 2018). The SEM analyses confirmed these expectations (Hypothesis 2a), in line with both neo-assimilation theory (Alba, 2008) and social identity theory (Tajfel & Turner, 1985). However, a weak interaction effect between the two orientations indicated that the positive relationship between host identification and academic performance may diminish if heritage orientation is high; on the other hand, there will be no accumulation of risks if host identification is weak and heritage orientation is strong. This does not exclude the possibility that heritage identification mediated by higher self-esteem also exerted a positive effect on academic performance, as suggested by Rivas-Drake et al. (2014). However, any such effect was negligible. These findings contradict the Additive Resource Model of biculturalism: In terms of academic performance, biculturalism did not appear to be an additional asset, but rather the second-best solution.

Social adjustment was assessed in terms of school attachment. As one of the main socialization agencies in adolescence, school exemplifies the normative expectations of the majority society. As the proximal social environment of a multiethnic student body, it is a place where young people of different cultural origins mix. Thus, school requires adolescents with immigrant background to learn to navigate multiple cultures, a competence that is likely to support motivational and emotional attachment to school (Schwartz et al., 2014). As predicted by Hypothesis 2b and consistent with previous empirical findings (Abu-Rayya & Sam, 2017; Schachner et al., 2017), the results confirmed the predictions of the Additive Resource Model, with host identification being more important for school attachment than heritage identification (*β*_*HOS*_ = 0.33; *β*_*HER*_ = 0.13). Biculturalism represented a cumulative resource; distance to both cultures, a developmental risk. A statistically significant negative interaction between host identification and Turkish origin indicated that the relatively strong positive relationship between host identification and school attachment was attenuated in this group. One reason could be that young people of Turkish descent—the largest immigrant group—formed a strong within-group that made identification with the host culture less relevant. Overall, school attachment was largely independent of individual background characteristics and the institutional context of the school.

Psychological adjustment was assessed in terms of self-esteem and life satisfaction. Consistent with self-categorization theory (Turner & Reynolds, 2011) and previous findings (Rivas-Drake et al., 2014) heritage identification proved to be an adaptive resource for self-esteem. This is in line with Hypothesis 2c. However, as the same did not apply to host identification, the present findings did not confirm the Additive Resource Model of biculturalism. The fully specified model showed that this finding was likely to hold across cultures of origin and immigrant generations. Unlike school attachment, self-esteem proved sensitive to the institutional context of the school attended. It increased with the proportion of adolescents with immigrant background in the grade cohort, but decreased with growing ethnic heterogeneity. This indicates that as an ethnic community grows within a school, the self-esteem of its members increases. Surprisingly, endorsement of a multiculturalism climate among majority peers was associated with decreased self-esteem among adolescents with immigrant background. However, this counterintuitive finding is consistent with critiques of the multiculturalism concept (Rios, 2022) and experimental findings by Wilton et al. (2019), who reported increased negative self-stereotyping of minorities when ethnic categorizations were accentuated in a multiculturalism climate (Baumert et al., 2023).

Likewise, the findings for life satisfaction did not support the Additive Resource Model of biculturalism. Here, however, significant functional differences in cultural identification emerged between the immigrant generations. In the first generation, only host identification seemed to support life satisfaction. In the second generation, both cultural orientations emerged as resources that were mutually substitutable (see Fig. 2c). In accordance with the compensation model (see Introduction; Rudmin, 2009; Ward & Kus, 2012), secure identification with either the heritage culture or the host culture was sufficient to stabilize life satisfaction. Dual cultural distance, however, proved to be a cumulative risk. Like self-esteem, life satisfaction emerged to be context sensitive: It increased with the proportion of immigrant background youth in the grade cohort, but decreased as the ethnic heterogeneity of the student body increased. Life satisfaction was unrelated to the school’s diversity climate. This is not to say that adolescents with immigrant background did not experience discrimination, microaggressions, or acculturation stress, which have repeatedly been shown to threaten well-being (Meca, Cruz, Lucero, et al., 2023; Schmitt et al., 2014; Zeledon et al., 2023). However, their on average strong heritage identification (see Table 3) could have buffered against such negative experiences (Litam & Oh, 2022; Oh et al., 2023; Shamloo et al., 2023).

A major strength of this study is that it simultaneously analyzed the relationship between cultural identification and the development of adolescents with immigrant background in four major dimensions of adjustment in a sample of 15–17-year-olds approaching the end of compulsory schooling. The study’s methodological strengths include its large random sample from a metropolitan area, the response rate of 94%, a design with two measurement points one year apart, rigor in controlling for potential individual and institutional confounders, and the latent modeling of cultural identification and adjustment with multiple indicators.

However, the study also has limitations in terms of content and methods. The most important content limitations probably relate to the instrumentation. Cultural orientations were captured solely in terms of commitment to the heritage and host culture. A multidimensional approach that takes into account the developmental process (e.g., exploration, resolution), centrality of dimensions, or cultural practices might yield different findings. Likewise, it is unfortunate that experiences that threaten self-esteem and subjective well-being, such as discrimination, microaggression, and acculturation stress (Zeledon et al., 2023), were not assessed. This limited the analysis of potential buffering functions of heritage identification. Recent studies have shown that a secure heritage identity can moderate threatening experiences, even in emergencies such as the COVID-19 pandemic (Litam & Oh, 2022; Oh et al., 2023; Shamloo et al., 2023). Another limitation to the generalizability of the findings is the sample, which comes from a metropolitan area of a single country. These limitations emphasize the need for replication and highlight challenges that should ideally be addressed in future studies.

Methodological limitations pertain primarily to the observational design of the study, which means that unobserved heterogeneity cannot be ruled out despite careful control of potential confounders. Caution is therefore warranted in any causal interpretation of the findings. Another limitation is that cultural identification and adjustment were assessed one school year apart, but that no baseline measure of adjustment was available. Ideally, a baseline assessment should occur at the beginning of secondary education, making it possible to capture the long-term process of adjustment. The present design has some merit over cross-sectional studies, but does not eliminate the possibility of interactions between cultural identification and adjustment prior to the first measurement point.

## Conclusions

As Western societies become more ethnically and culturally diverse, understanding the acculturation of immigrant youth is essential for fostering social cohesion. This study contributes two main findings that advance this understanding. First, patterns of cultural identification among adolescents with immigrant background did not differ systematically with perceived distance from the majority culture, reason for migration, Islamic heritage, or skin color. To a certain extent, cultural identity formation seemed to be protected against the influence of integration-hostile attitudes among the adult population of the host country. It is possible that the multicultural climate of public schools—at least in metropolitan areas with a large and diverse immigrant population such as Berlin—serves a buffering function here. Second, the implications of identifying with the heritage or the host culture differed markedly depending on the dimension of adjustment. This domain specificity of findings challenges the generalization claims of predominant acculturation models, in particular biculturalism theory, and poses a conundrum with regard to practical interventions and integration policies: Host identification was beneficial for academic performance and school attachment, and it could even substitute for heritage identification with regard to life satisfaction. It was never a risk factor. Heritage identification was beneficial for school attachment, self-esteem, and life satisfaction, serving as a resource that may protect against experiences such as discrimination, microaggression, and acculturation stress. However, it was a potential risk factor for academic performance. Against this background, supporting biculturalism—which is in fact the modal form of acculturation of adolescents—seems to be not a panacea, but potentially a wise compromise: Acknowledging and nurturing both the host and heritage cultural identities of immigrant youth can lead to better outcomes in terms of overall adjustment.

### Supplementary information

Appendix
