# Supplementary material for: Cultural Identity and the Academic, Social, and Psychological Adjustment of Adolescents with Immigration Background
Source: J Youth Adolesc. 2023 Sep 16;53(2):294–315. doi: 10.1007/s10964-023-01853-z (PMC10764554; doi:10.1007/s10964-023-01853-z)
Supplement: Supplementary file 1 — Appendix [file 10964_2023_1853_MOESM1_ESM.docx]

**Appendix**

| **Table A1**  *SEM Analyses: Regression of Academic Performance, School Attachment, Self-Esteem, and Life Satisfaction on Cultural Identification: Separate and Concurrent Estimations (Standardized Regression Coefficients, Standard Errors in Parentheses,* N*= 833)* | | | | |
| --- | --- | --- | --- | --- |
| Predictors | Academic performance^a^ | School attachment^b^ | Self-esteem^c^ | Life satisfaction^d^ |
| *Separate analyses* |  |  |  |  |
| Identification host culture (HOS) | 0.183** (0.052) | 0.259** (0.05/) | −0.023 (0.061) | 0.171* (0.068) |
| Identification heritage culture (HER) | −0.144** (0.057) | 0.130* (0.060) | 0.114** (0.049) | 0.204** (0.065) |
| Interaction HOS × HER | −0.152** (0.054) | −0.058 (0.065) | −0.050 (0.067) | −0.265** (0.049) |
| *Concurrent analyses without life satisfaction* |  |  |  |  |
| Identification host culture (HOS) | 0.187** (0.050) | 0.264** (0.057) | −0.032 (0.063) | 0.171* (0.068) |
| Identification heritage culture (HER) | −0.138** (0.056) | 0.127* (0.060) | 0.117* (0.053) | 0.204** (0.065) |
| Interaction HOS × HEC | −0.167** (0.056) | −0.061 (0.063) | −0.034 (0.077) | −0.265** (0.049) |
| *Concurrent analyses* |  |  |  |  |
| Identification host culture (HOS) | 0.185* (0.049) | 0.263** (0.057) | −0.056 (0.069) | 0.201** (0.062) |
| Identification heritage culture (HER) | −0.134** (0.055) | 0.135* (0.060) | 0.077 (0.062) | 0.139* (0.045) |
| Interaction HOS × HEC | −0.159** (0.048) | −0.060 (0.054) | 0.069 (0.111) | −0.161** (0.045) |

^a^ Latent construct; measurement model: final grades in German, the first foreign language, math, physics, and biology at the end of grade 10.

^b^ Latent construct; measured with eight indicators (see Methods).

^c^ Latent construct; measured with four indicators (see Methods).

^d^ Latent construct; measured with four indicators (see Methods).

***p* ≤ .0.01, **p* ≤ .05, ^+^*p* ≤ .10.
